# Supplementary material for: Genomic Analysis of the Suspicious SARS-CoV-2 Sequences in the Public Sequencing Database
Source: Microbiol Spectr. 2023 Jan 9;11(1):e03426-22. doi: 10.1128/spectrum.03426-22 (PMC9927258; doi:10.1128/spectrum.03426-22)
Supplement: Supplemental file 1 — Supplemental material. Download spectrum.03426-22-s0001.pdf, PDF file, 1.3 MB [file spectrum.03426-22-s0001.pdf]

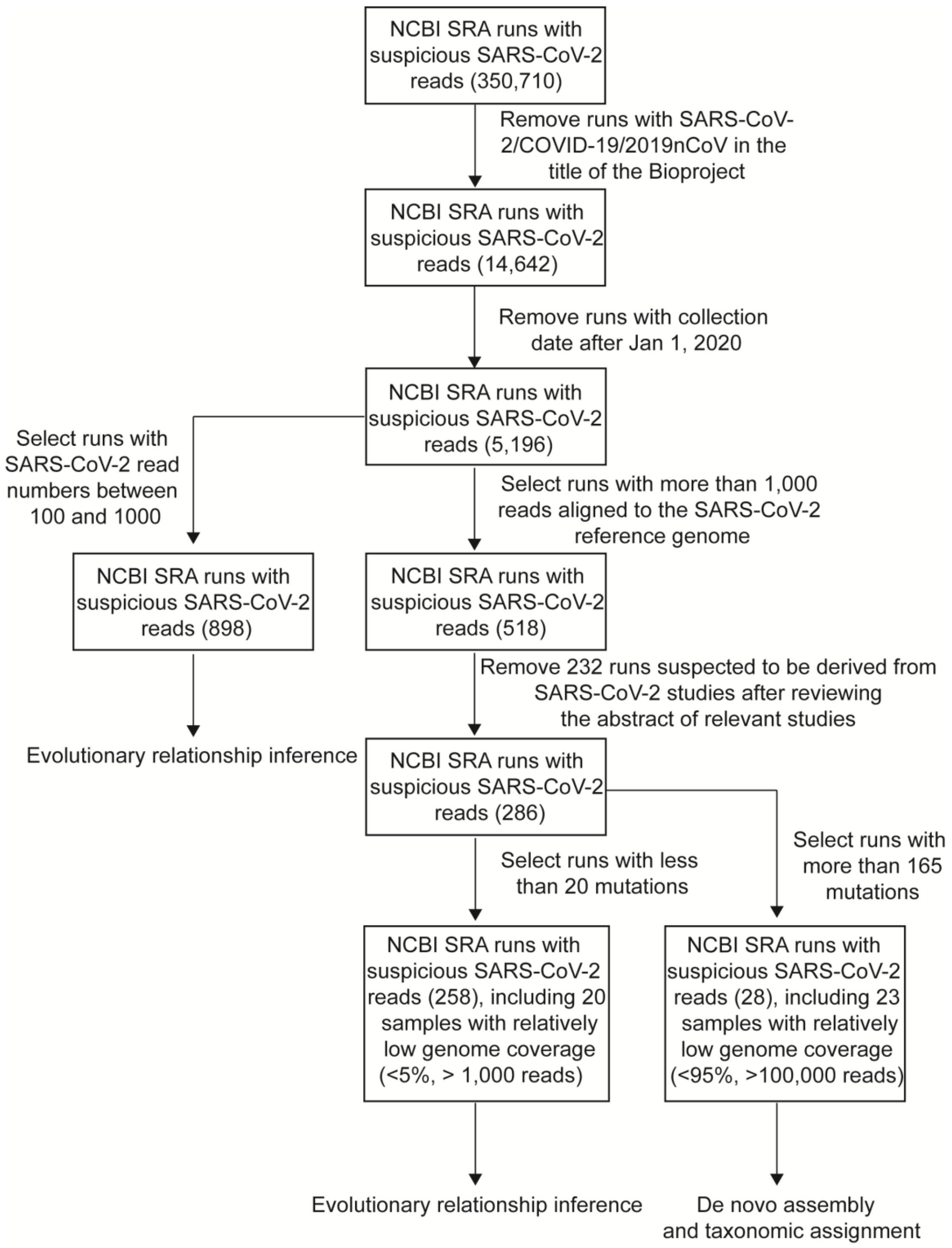

Supplementary Figure 1. The flowchart of data filtering and analysis.

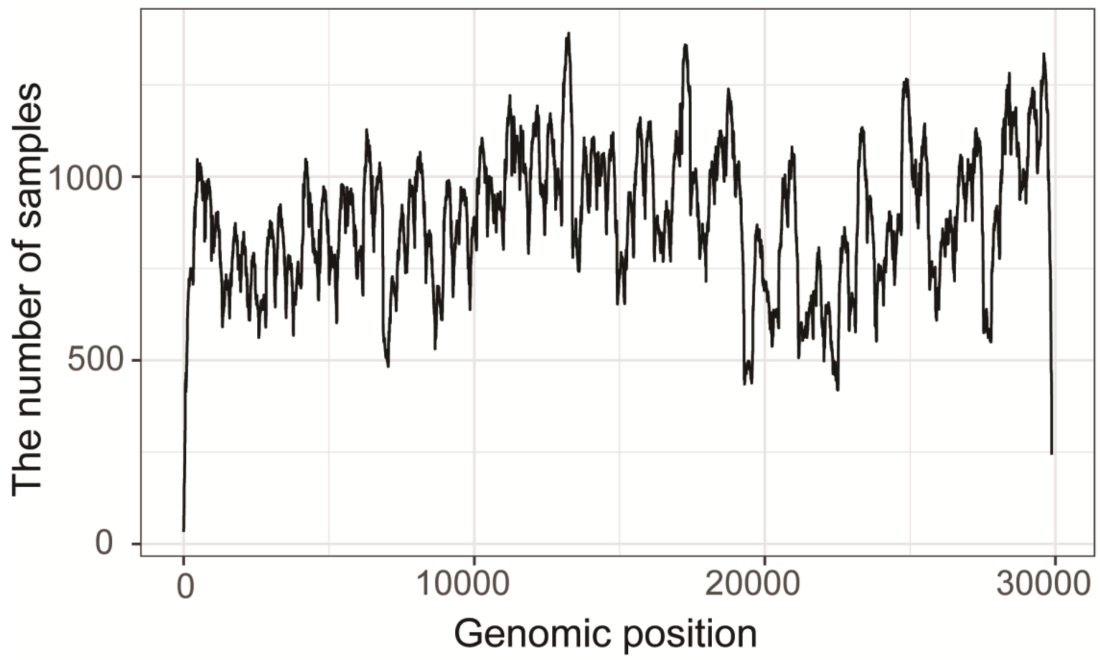

Supplementary Figure 2. The coverage of the SARS-CoV-2 genome among 5196 suspicious samples. The number of samples with reads aligned to a specific region on the SARS-CoV-2 genome is shown on the Y-axis.

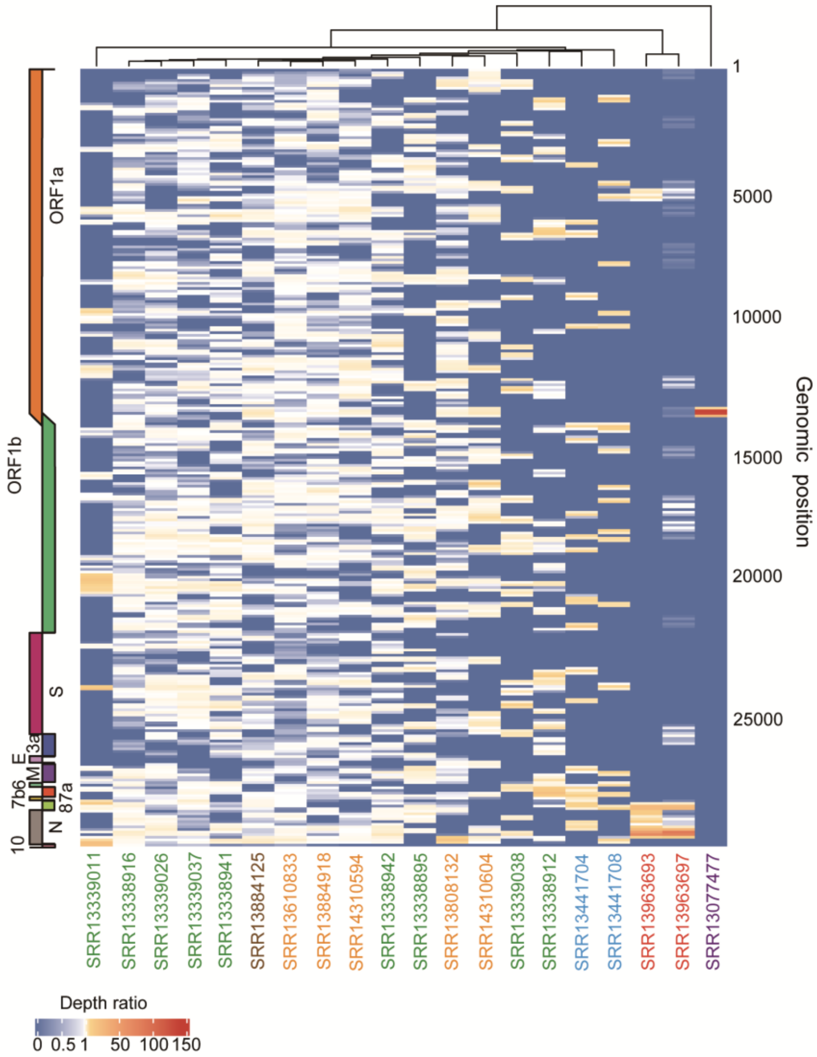

Supplementary Figure 3. The sequencing depth on the SARS-CoV-2 genome of 20 samples with low genome coverage. Only samples having more than 1000 SARS-CoV-2 reads and less than 5% of the genome covered by at least five reads were included in the analysis. The sequencing depth was calculated for every 100bp on the genome without overlap and then normalized by the average sequencing depth on the complete genome. Accession numbers from the same study are labelled with the same color.

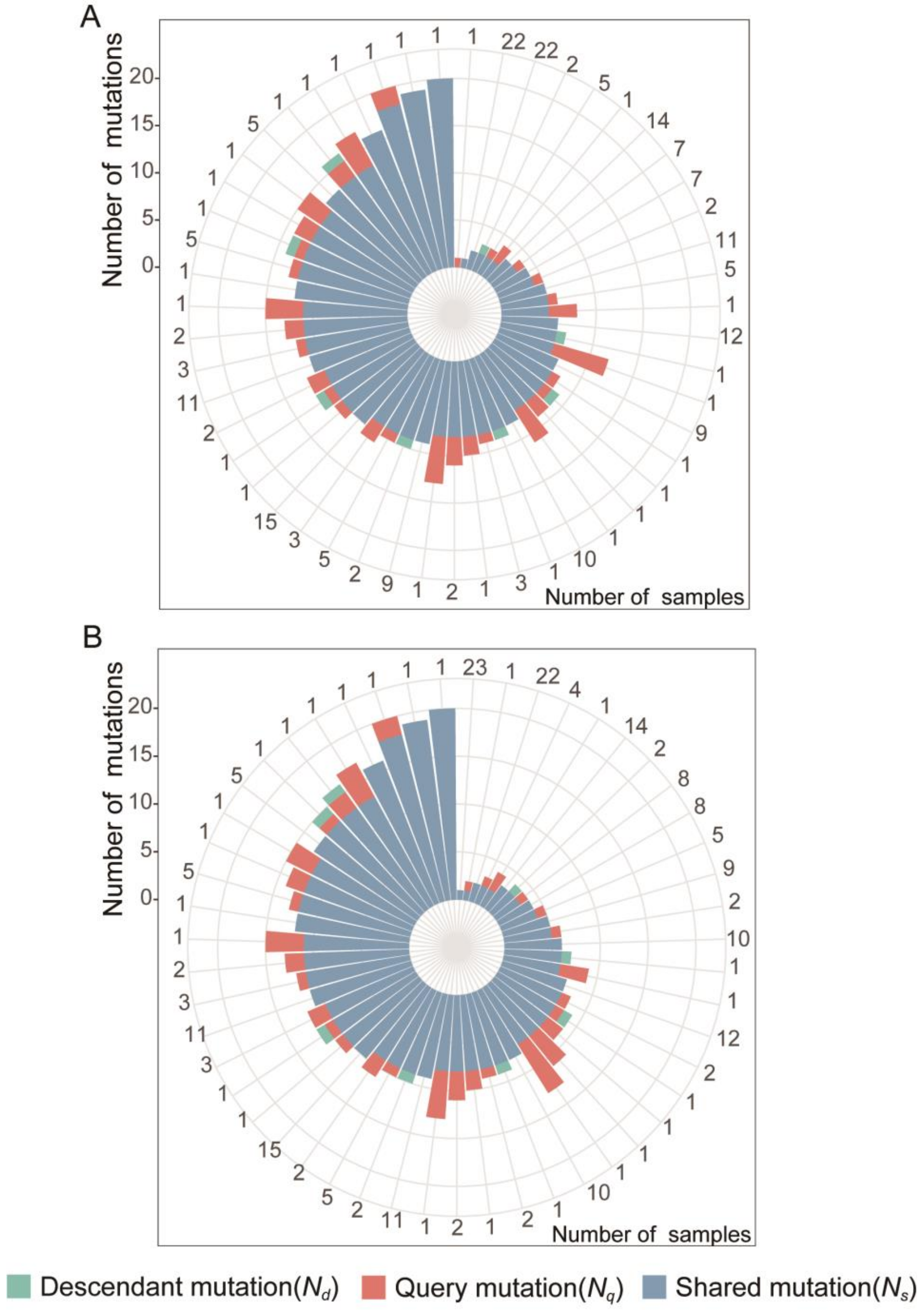

Supplementary Figure 4. Distribution of the number of different mutation types for 286 questioned sequences. A. Number of mutations when ProCoV2 was used as the earliest SARS-CoV-2 sequence (E). B. Number of mutations when Guangdong/HKU-SZ-002/2020 was used as the earliest SARS-CoV-2 sequence (E). Samples with no mutations relative to the earliest SARS-CoV-2 sequence were not included in the figure.

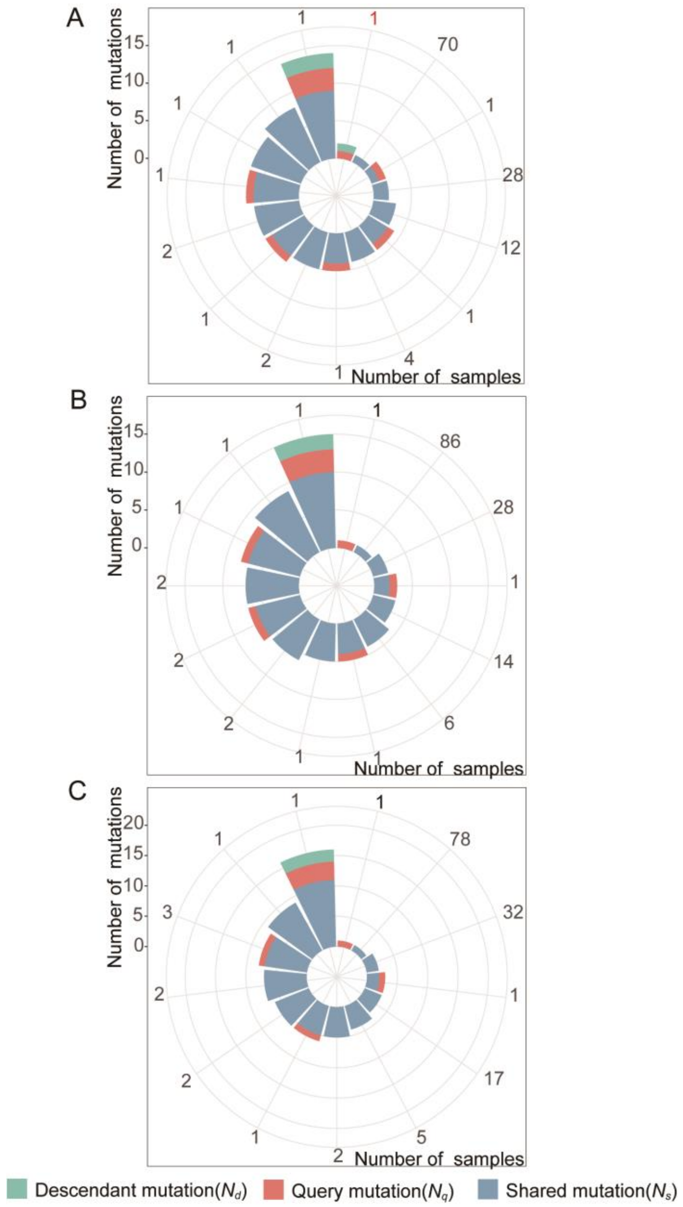

Supplementary Figure 5. Distribution of the number of different mutation types for 898 questioned sequences (with SARS-CoV-2 read number between 100 and 1000). A. Number of mutations when NC_045512.2 was used as the earliest SARS-CoV-2 sequence (E). B. Number of mutations when ProCoV2 was used as the earliest SARS-CoV-2 sequence (E). C. Number of mutations when Guangdong/HKU-SZ-002/2020 was used as the earliest SARS-CoV-2 sequence (E). Samples with no mutations relative to the earliest SARS-CoV-2 sequence were not included in the figure.

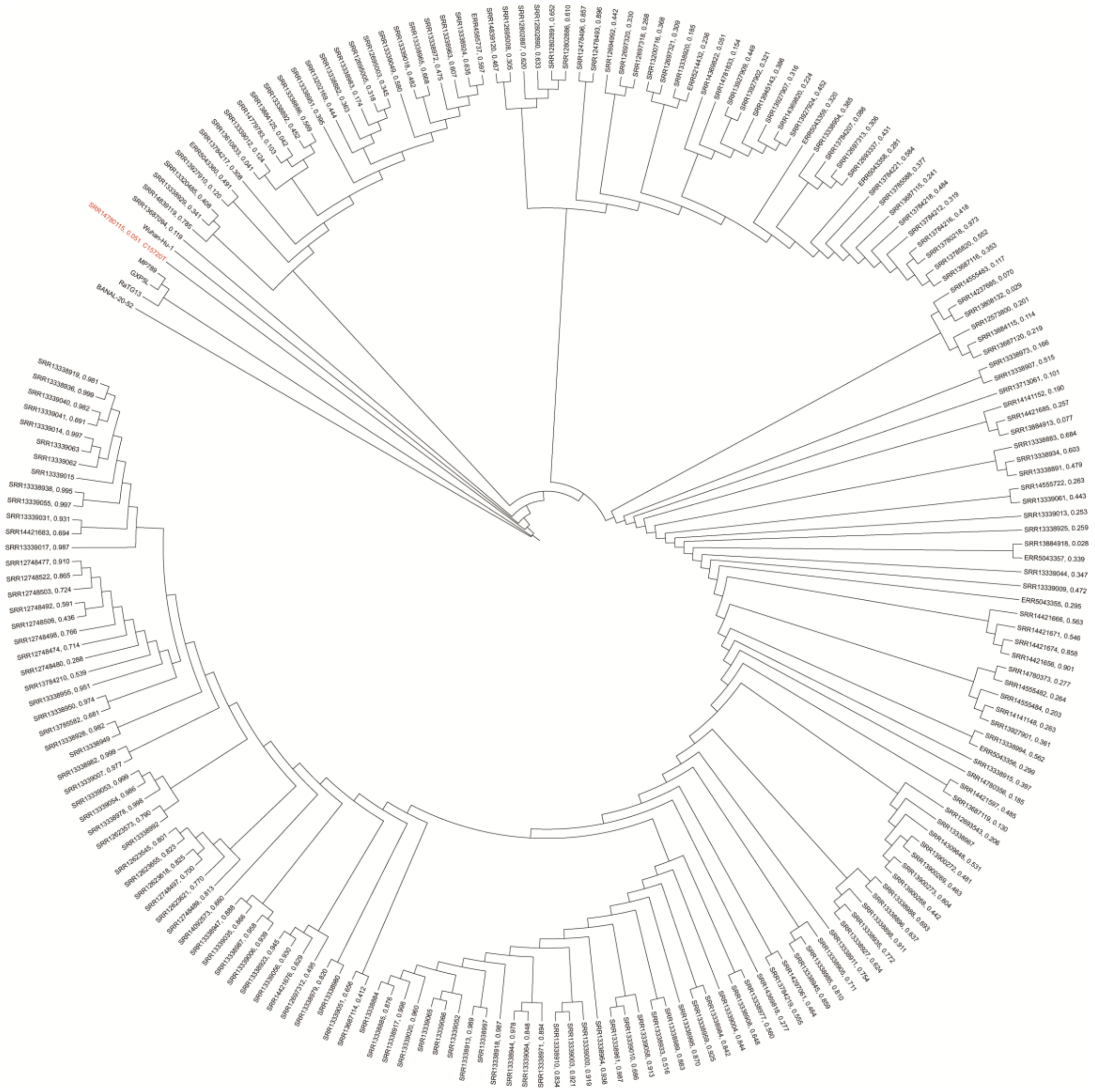

Supplementary Figure 6. The phylogenetic tree constructed using all suspicious SARS-CoV-2 sequences and putative ancestral sequences of SARS-CoV-2. The uncovered genomic regions were filled with the SARS-CoV-2 reference genome (NC_045512.2). The sequences of four putative distant ancestors of SARS-CoV-2 (RaTG13, GXP5L, MP789, and BANAL-20-52) together with a known earliest SARS-CoV-2 sequence (NC_045512.2) were included in the analysis. The accession numbers of sequences and the genomic coverage of the sequence are shown in the figure. The accession number of the suspected progenitor sequence is marked in red.
